# Development of water insecurity scale for rural households in Cameroon- Central Africa

**DOI:** 10.1080/16549716.2021.1927328

**Published:** 2021-06-24

**Authors:** Carole Debora Nounkeu, Kenneth J. Gruber, Joseph Kamgno, Ismael Teta, Jigna Morarji Dharod

**Affiliations:** a Limbe Regional Hospital, Limbe, South-West, Cameroon; b Center for Housing and Community Studies, The University of North Carolina at Greensboro, Greensboro, NC, USA; c Faculty of Medicine and Biomedical Sciences, University of Yaoundé I, Yaoundé, Cameroon; d Director of the Centre for Research on Filariasis and other Tropical Diseases (CRFilMT), Yaoundé, Cameroon; e Hellen Keller International Cameroon, Yaoundé, Cameroon; f Department of Nutrition, University of North Carolina at Greensboro, Greensboro, NC, USA

**Keywords:** Food insecurity, water, diarrhea, sustainable development goals, gender equity, nutrition

## Abstract

**Background**: Water represents the core of food-energy nexus and is vital for human survival. In developing countries, contaminated water and lack of basic water services undermine efforts to improve nutritional status and related health issues. In the rural areas of Central Africa, a majority of the population lacks access to improved water sources and has to devote considerable efforts to obtain water.

**Objectives**: Using the following definition of water insecurity, i.e. it exists when access to adequate amount of safe and clean water does not occur all the times for the entirety of household members to lead a healthy and active life, the study aimed to develop and test a household-level experiential water insecurity scale for rural households in Central Africa.

**Methods**: The research was conducted in three phases: 1) the formative data collection; 2) the scale development; and, 3) the scale testing. In the third Phase, the scale was tested with 250 women who were water managing person of their households. Statistical analysis included items reduction, reliability, as well as criterion and construct validity assessment. The testing led to a final scale of 17 statements (WATINE-17), covering three domains of water insecurity: 1) psychosocial distress; 2) quantity; 3) quality of water.

**Results**: The scale showed an excellent reliability (Cronbach’s alpha = 0.92) and was significantly associated with lower frequency of water intake among women (p = 0.007, concurrent validity). In assessing WATINE-17’s predictive validity, it was found that water insecurity was positively related to food insecurity (p < 0.001) and the level of water insecurity was the highest among severely food insecure households [F (3, 246) = 22.469, p < 0.001].

**Conclusion**: The WATINE-17 is able to capture key elements of water insecurity and can be used to monitor and evaluate SDG# 6 and water-related programs, such as WASH, in Central Africa.

## Background

In 2010, the United Nations (UN) recognized the access to clean drinking water as a basic human right. However, approximately 844 million people worldwide still lack basic water services and 2.1 billion do not have access to clean drinking water [1]. From a nutritional perspective, the use of unsafe water and poor sanitation increases the incidence of repeated diarrhea and intestinal worm infections, which can result in inadequate food utilization and improper nutrient absorption, especially among children. In fact, in developing countries it is estimated that approximately 840,000 deaths per year occur due to illnesses and diseases attributed to poor sanitation and hygiene resulting from limited access to clean water [2]. Diarrheal diseases are the most common manifestation of waterborne infections and the second leading cause of death among under-five children [2]. Poor environmental conditions due to unsafe water cause chronic intestinal inflammation and poor absorptive function, leading to stunted growth and decreased cognitive development among children [3]. These and other conditions motivated the recognition by the UN of the importance of water in improving health and maintaining a strong ecosystem. Subsequently, one of the 2030 Sustainable Development Goals (SDG) is to achieve universal access to clean water and sanitation (SDG# 6) [1].

In 2013, the UN-Water’s Integrated Monitoring Initiative adopted a definition for the water security and established distance and time-related indicators to measure it at the national, regional, and community levels [4,5]. Water security is considered at risk when the improved water source is not within 1000 m of the home (distance indicator) and/or total water collection takes more than 30 minutes (time indicator) [5]. Access to life sustaining water however, cannot just be measured in terms of time and distance. Water is an essential nutrient and its sufficient daily intake is vital for the human physiology and optimal health. Hence, a robust measurement is needed to understand how water insecurity is associated to daily water use and intake at the household and individual level [6]. Water is also a key component for food production and plays a key role as a critical factor affecting food security for smallholder farmers in rural areas [7,8].

Several studies have been conducted to develop a household-level water insecurity scale [9–17]. However, a review examining the current status of research on water insecurity measurement indicates that the scale development and testing lacks uniformity and reveals a gap in the identification of key water insecurity domains [6]. Learning from the household-level food insecurity scale development, multiple efforts are needed involving ground-up development and testing in various settings to refine and establish a reliable water insecurity scale with high external validity. Furthermore, recognizing water: 1) as an essential nutrient and how water insecurity affects its intake related behavior; and 2) acknowledging the importance of water in ensuring the three pillars of food security, i.e. food production, access, and utilization, we determined that a more comprehensive water security scale was needed.

Thus, drawing upon extensive and well-established argument on the importance of measuring food insecurity at the household-level [18,19], we undertook the development of a validated household-level experiential water insecurity scale using its pre-determined definition. We focused the scale on the concept that in developing countries, caretakers or female adults play a primary role in fetching and managing water for their households, including cooking and feeding infants and children, ensuring food availability and utilization for their families [8,15,20]. International agencies such as the International Fund for Agricultural Development (IFAD) and the UN have recognized the need to empower women in order to address water and food insecurity. This scale development study was conducted involving validity testing of indicators of food insecurity, daily water intake and frequency of water intake among female caretakers, who typically were primary persons involved in water fetching and management at the household level.

## Methods

### Study area

The study was conducted in Cameroon, a Central African country, also called ‘Africa in miniature,’ due to its multiple ethnic groups that are also found in most of African countries and its high linguistic and geographical diversity [21,22]. The study specifically took place in the Menoua Division, West Region – Cameroon, which is divided into 22 villages, with Bamileke being the main tribe represented. The main religion is Christianity and more than 80% of the population are farmers. The Menoua Division is part of the French-speaking portion of the country. French is spoken more commonly than English. There are two main seasons in the study area, – (1) a dry season from November to March and (2) a rainy season in the remaining months (annual rainfall on average is about 71 inches) [21,22]. The selection of this area for the study was based on our previous collaboration with the Division Officials and its representation of rural settings in Central Africa where economic water scarcity prevails despite the physical availability of freshwater [21–24].

### Study design

#### Overall description of the study design

**Figure 1.**
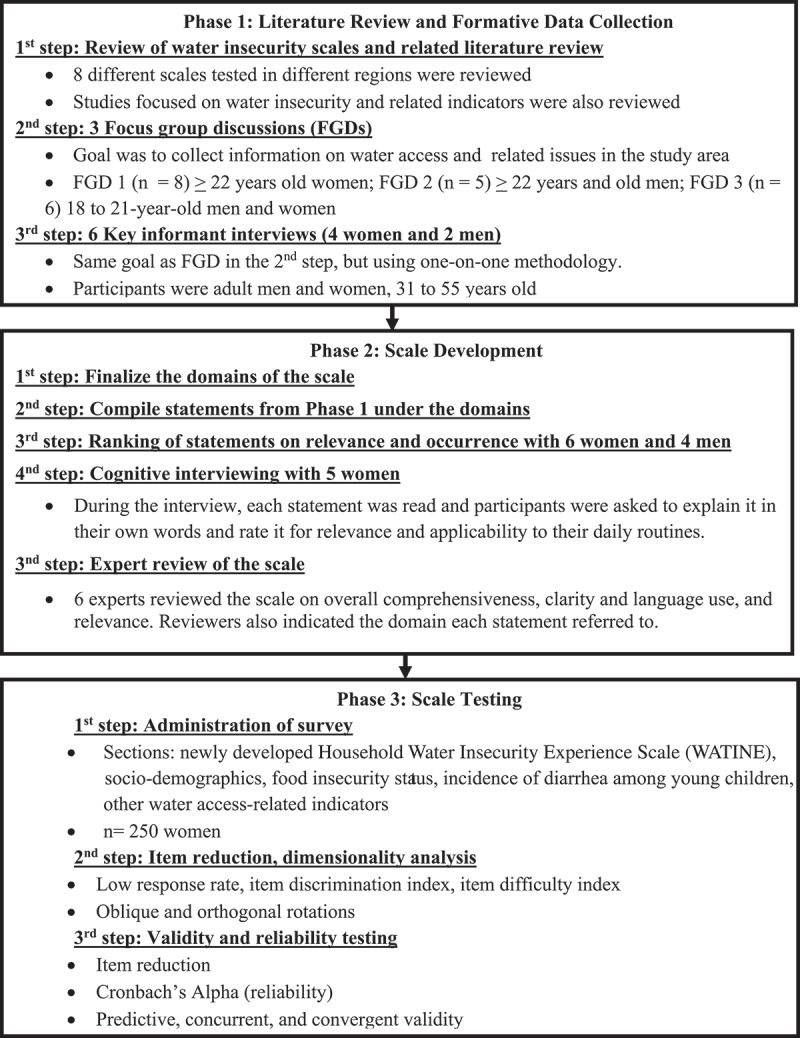
Description of steps involved in the ground-up development and validation of the household water insecurity experience scale (WATINE) conducted in rural areas in West Cameroon – Central Africa

Approvals were obtained from the Cameroon’s National Committee of Ethics and the University of North Carolina at Greensboro’s Institutional Review Board. The study was conducted from February 2019 to January 2020, in three phases. As shown in Figure 1, Phase 1 consisted of literature review and formative data collection. The first and foremost step was to review existing scales and literature on water insecurity to identify potential topics of enquiry and design questions for the formative activities including focus group discussions (FGDs) and key informant interviews (KII) with adult men and women from the study population. The goal of this phase was to collect information on water access and identify issues, concerns, and water-related coping strategies people practiced in the study area. In Phase 2, the domains of the scale were finalized, and statements generated from Phase 1 were compiled under each of the domains. The statements under each domain were then ranked for adult women and men from the study area. Based on the ranking results, the first draft of the scale was developed. Cognitive interviews were conducted with five women for the purpose of revising and improving the wording of each item in the scale., The revised scale was then reviewed by experts in the field of scale development, water insecurity, and/or public health regarding its overall comprehensiveness. They were asked to rate each statement for clarity, language use, and relevance to the following water insecurity definition adopted for the study: *Water insecurity refers to when the access to adequate amount of safe and clean water does not occur all the times for the entirety of household members to lead a healthy and active life*.

Lastly (Figure 1) in Phase 3, scale testing was carried out. During this phase, statistical tests were applied for item reduction and dimensionality of the scale. For validity assessment, the scale was compared with the food security status and other water access-related indicators. Information on socio-demographics and daily water intake were also collected during the survey. All the study activities in Phase 1 as well as ranking of items and cognitive interviews in Phase 2 were conducted in French. For the analyses of these activities, all the transcripts and open-ended data were translated into English by a bilingual primary author and the translations were randomly cross-verified by a third person fluent in English and French but not part of the research team. The expert reviews in Phase 2 involved both English and bilingual speakers to ensure the scales across the two languages were essentially the same. The activities in Phases 1 and 2 were carried out by first author of this manuscript, a doctoral student at the time, and Co-Principal Investigator of the project. Along with extensive experience in conducting qualitative interviews, first author had an established close relationship with the study community due to previous research in the study area.

## Methodology of the data collection

### Phase 1: literature review and formative data collection

Based on the findings from previous water access-related studies conducted in the study area [25,26] and existing household water insecurity scales literature [6], questions for the FGDs were designed.

As the first step, two FGDs were conducted with women (n = 8) and men (n = 5) separately. Women were selected due to their involvement in water fetching and water use management in their houses. To get a full perspective, FGDs were also conducted with men, who were head of the households. The discussions were carried out to gather information on water sources, water fetching, water management, daily water use, and water-related roles as well as responsibilities among household members. Questions were also asked to gather information on concerns and common strategies used to manage limited water. Data on seasonal variations, i.e. dry vs. wet season also were collected. Based on the results of these two FGDs, it was established that young adults also played a key role in water fetching and management besides women in the households. So, a third FGD took place involving 18 to 21 years old boys and girls (n = 6) selected from different households of the community. All the FGDs were conducted in French and each lasted for approximately 2 hours.

The next step in the process involved the key informant interviews (KII). The key informants included two men and four women well-established in the community and from diverse professional background: teachers, engineers, sellers, and farmers. The main goal of these in-depth interviews was to confirm and obtain more significant details on water-related issues and strategies used, in addition to corroborating daily water fetching and management-related routine in the study area. Results of this formative phase are published elsewhere [8].

### Phase 2: scale development

Based on the definition agreed upon by the authors and the results of Phase 1, three domains for the scale were identified: 1) psychosocial distress due to inconsistent or limited access to water; 2) water quantity, and 3) water quality. All the relevant statements from Phase 1 were compiled under the three domains, leading to a list of 81 statements for ranking using the choice option scale approach and following the Likert design with three levels. The ranking of items was conducted with six women and four men randomly selected in the community and who did not take part to the FGD and KII. Each item was presented to the participants as a statement describing a water-related situation or experience that could be encountered in their day-to-day activities. They were read each statement and asked to rate each one on relevance (very much, somewhat, not at all) applicability in reference to their daily routine. The ranking of item analysis was conducted progressively and the interviews were stopped when saturation was reached in answers. This ranking step resulted in short-listing of statements to 40 (see Figure 1).

Then as a next step, cognitive interviews were conducted with five women (Figure 1) by the first author. Each participant was read aloud each individual statement and asked to explain it in her words. Participants were also asked to select statements by relevance and applicability to their daily routines. For each statement, a reference period of past 4 weeks was used with response options of yes and no, and if yes, the follow-up question of how frequently was asked. The frequency options provided were: rarely = once or twice in the past 4 weeks, sometimes = three to ten times in the past 4 weeks, and often = more than ten times in the past 4 weeks. Both time reference period and options were also discussed and tested during the cognitive interviews. In the end, the answers from the respondents were put side-by-side to facilitate the summary process and examination of commonalities in participants’ responses. Statements that were deemed highly relevant by the respondents were also checked for wordings and clarity. Those judged as not representing a common experience were removed. This step led to the reduction into 30 statements of the household water insecurity scale (WATINE) or in a short form, the WATINE-30.

The WATINE-30 was then reviewed by five subject experts and one bilingual teaching professional (see Figure 1). Of the five subject experts, two reviewed the scale in French, and the remaining three in English. They were specialized in the area of household water insecurity, scale development, and/or public health. Along with the scale, the expert reviewers were provided with the water insecurity definition agreed upon by the research team after Phase 1, three domains of water insecurity, and the guidelines on rating the WATINE-30. All the five subjects experts were requested to rate each statement on a scale of 1 to 4 as concerning clarity/language use and relevance. Reviewers were asked to provide reasoning for any low ratings of 1 or 2 and suggestions if any on how to improve those statements. They also were asked to attribute a domain for each of the statements. The bilingual teacher proficient in English and French languages specifically reviewed the scale in the both the languages to compare and ensure that the statements were identical between English and French scale.

For the analyses, Modified Kappa statistics (K) values were used to assess the relevance of each of the statements related to the water insecurity definition. The Kappa value was calculated based on the rating provided by all experts for each item and interpreted as follows: K > 0.74: excellent; 0.60–0.74: Good; 0.40–0.59: Fair. All the statements yielding an excellent Kappa value were retained in the scale and those with a value < 0.4 considered for deletion. Also, the inter-rater agreement value, i.e. the count of number of experts who rated 3 or 4 for the item divided by the total number of items was used to evaluate the overall clarity of the scale. The results were interpreted following the present thresholds: 0–0.30: lack of agreement; 0.31–0.50: weak agreement; 0.51–0.70: moderate agreement, 0.71–0.90: strong agreement, 0.91–1.0: very strong agreement [27–30]. Results from this step led to a reduced version of the scale with 25 statements, the WATINE-25-25.

### Phase 3: scale testing

The WATINE-25-25 was then administered using a cross-sectional study design to a sample of 250 women who were primarily responsible for managing water fetching for their households. The sample size was estimated on the basis of the general thumb rule of 10:1 ratio, i.e. having at least 10 participants per scale item [27]. In addition to the scale, the survey also included the following sections for analyses and validity testing: 1) socio-demographics; 2) food insecurity assessment using the FAO’s Household Food Insecurity Access Scale (HFIAS) [31]; 3) water access-related indicators, i.e. time spent to fetch water involving walking to and from the water source and queuing, amount of water stored in the household at the time of the survey, and the total amount of water used by the household in the past 24 hours. Women also were asked questions to estimate their daily intake of water in the past 24 hours, including the frequency of the intake. Household with children born between 2014–2019 (5 years or younger age) were asked if the youngest child in the household had diarrhea in the past 4 weeks (Yes or No).

Participants were recruited using the snowball sampling technique and door knocking approach. The survey was conducted in participants’ households by the primary author or research assistants either in French or in Yemba (a local dialect). Research assistants were college students from the nearby university, fluent in French and Yemba. In interviews in Yemba, the survey in French was translated in real-time by the interviewers. Prior to the survey, several training sessions were conducted to train the team of research assistants on interview techniques and data collection procedure.

The survey data was analyzed using SPSS 23 (IBM Corp, NY). Table 1 provides information on step by step procedure and testing to develop the WATINE-25 and test it for reliability and validity. Specifically, item reduction was carried-out using non-response rates, item difficulty index, and item discrimination index. All the statements with: (1) high missing cases (>10%); (2) low (<30%) or high (>80%) difficulty index; and/or, (3) low (≤0.14) or high (≥0.86) discrimination index were removed from the scale. Then, an exploratory factor analysis was conducted using both orthogonal (varimax) and oblique (direct oblimim) rotations to determine the number of latent factors that fitted the data. All the statements not meeting the adopted thresholds (factor loading of 0.40 and 0.50 respectively) or that cross-loaded (loaded more than the threshold on two or more factors) in both rotations were dropped from the scale. The Cronbach’s coefficient alpha was used to assess the reliability of the scale with a value of >0.80 considered acceptable and >0.90, excellent reliability. Finally, criterion and construct validity assessments were carried out to determine the predictive, concurrent, and convergent validity of the scale. The p-value for significance was ≤0.05 and for marginal significance ≤0.1.

**Table 1.** Summary of the step by step procedure and tests that were carried out during the phase 3 involving testing of the household water insecurity experience  scale (WATINE) in rural areas in West Cameroon-Central Africa (n = 250)^†^

Step	Purpose	Methodology	Outcome
Item Reduction and Dimensionality Analyses			
Response rates	Assess response rate of participants to each statement	Percentage of participants answering no or not applicable to each statement Statements with >10% non-response rate considered for deletion	
Item difficulty index	Determine whether the statement is too easy or too hard for the participants to rightly associate them to their water insecurity experience	Defined as the proportion of answers coded ≥1 for each statement Statements with percentages ≤ 30 or ≥80 considered for deletion	Led to reduction of HWINS into 21 items
Item discrimination index	Determine the ability of the statements to separate participants based on their water insecurity status	Corrected point-biserial correlation coefficient Statements with scores either ≥0.86 or ≤0.14 considered for deletion	
Exploratory factor analysis	Explore the latent components of statements	Direct quartimin and varimax rotation tested Statement with low factor loadings and cross loadings were considered for deletion	Led to reduction of WATINE to 17 item
Reliability and Validity Testing of WATINE 17			
Cronbach Alpha	Assess reliability of the scale	Calculated for the initial version of the scale as well as the reduced version of the scale alpha >0.80 acceptable; >0.90 excellent reliability	Excellent reliability α(WATINE-25) = 0.932 α(WATINE-17) = 0.923
Predictive validity	Test the ability of the scale to predict future water insecurity-related outcomes	Food insecurity score: Pearson correlation Food insecurity categories: One way ANOVA Diarrhea incidence among children 5 or younger age: one way ANOVA	The scale predicted food insecurity with a dose-response relationship The scale predicted the incidence of diarrhea in the past 4 weeks
Concurrent validity	Test how well the scale compares to well established standard	Reference daily water intake of 2.7 liters for women: Pearson Correlation	No correlation for daily intake Correlation for the frequency of intake
Construct validity	Test the correlation of the scale score to other constructs known to assess water insecurity	Time spent to fetch water: Pearson correlation Amount of water available in storage containers: Pearson correlation	Positive correlation

Testing of HWINS was carried out with 250 women who were main person involved in managing water fetching for the household.

## Results

### Analysis of the WATINE-25

The formative and scale development phases (Phases 1 and 2) led to the WATINE-25 (Table 2) covering three domains of water insecurity: 1) psychosocial distress due to inconsistent or limited water access, 2) quantity of water and, 3) water quality. The reliability and validity of WATINE-25 was tested by administering the scale to 250 women from the community who were responsible for securing water for their households.

**Table 2.** Progressive versions of the household water insecurity scale (WATINE) tested in rural areas in West Cameroon- Central Africa

WATINE-25^†^	WATINE-21	WATINE-17
Psychosocial distress due to inconsistent or limited water access		
S1. .were you dissatisfied with the water situation of your house?	S1. X	–
S2. .did you worry that there would not be enough water for your house?	S2. R	S2. R
S3. .were you upset because the water source dried up, or was not working, or water service was interrupted?	S3. R	S3. R
S4. .did you get into an argument or fight over getting water for your home?	S4. R	S4. R
S5. .did you have to pay or give food or flatter somebody to get some water for the house?	S5. R	X
S6. .did you borrow water from others because there was not enough water in the house and going to a water source was not an option?	S6. R	X
Quantity of water		
S7. .were you not able to properly finish your daily work/chores due to not having enough water ?	S7. R	X
S8. .were you not able to cook because there was not enough water in the house?	S8. R	S8. R
S9. .did you have to cook something different than you wanted to because there was not enough water in the house?	S9. R	S9. R
S10. .were you not able to wash dirty utensils because there was not enough water in the house?	S10. R	S10. R
S11. .were you not able to wash your dirty clothes on an habitual laundry day because there was not enough water in the house?	S11. R	S11. R
S12. .were you not able to clean yourself or take a bath because there was not enough water in the house?	S12. R	S12. R
S13. .were you not able to wash your hands when you wanted to because there was not enough water in the house?	S13. R	S13. R
S14. .were you not able to clean latrines/toilets because there was not enough water in the house?	S14. R	S14. R
S15. .did your children go to school late because water fetching took too long?	S15. X	–
S16. .did you miss farm work or your job work because of water issues?	S16. R	S16. R
S17. .did it occur that there was no water in the house and there was no way of getting water immediately?	S17. R	S17. R
S18. .did you stop raising pigs or other livestock, such as goats or chicken because there wasn’t enough water to take care of them	S18. X	–
S19. .did you stop cultivating/watering a home garden because there wasn’t enough water?	S19. X	–
S20. .did you wake up earlier than usual time to fetch water for the house?	S20. R	S20. R
WATINE-25^†^	WATINE-21	WATINE-17
Quantity of water – Specifically in reference to drinking or daily intake of water		
S21. .did you drink less than you feel you should because there was not enough water in the house?	S21. R	S21. R
S22. .did you sleep thirsty because there was not enough water in the house?	S22. R	S22. X
Water quality		
S23. .did you use the same water for multiple usages, such as same water to wash clothes, wash utensils, or wash hands and then wash something else?	S23. R	S23. R
S24. .did you drink muddy, unclear, or bad smelling water because there was not enough water in the house?	S24. R	S24. R
S25. .were you not able to drink water because it did not taste good?	S25. R	S25. R

^†^All the statements started with ‘in the past 4 weeks …’ R: Retained. X: removed at that version; – removed at the previous version.

The average age of the sample was 44 ± 16 years; about 44% of the participants were living in the household with 5 years or younger age children. The average household size was five, with about three of the family members being children (Table 3). Approximately half of the women had either elementary level or no formal schooling. Specific information on household earnings was not collected since living on a consistent income was not common in the study area. However, information to assets and wealth-related indicators showed that 66% of participants owned a livestock such as pig, chicken, or goats. In addition, 41% reported some type of flooring other than dirt and 80% used charcoal or wood for cooking.

**Table 3.** Socio-demographic characteristics, food security status, water fetching and water use among women in rural areas in West Cameroon-Central Africa (n = 250)

Socio-demographic and water-related characteristics	Mean ± SD^#^
Women’s age (in years)	44 ± 16
Total household size	5 ± 2
No. of children living in the household (<18 years)	3 ± 2
Time it took to fetch water per trip (in minutes) ^†^	37 ± 39
Amount of water available in the household (in liters) ^‡^	25 ± 33
Amount of water used by the household in the last 24 hours (in liters)	65 ± 42
	n (%)^Δ^
Education	
No formal schooling	32 (13)
Elementary school	93 (37)
Middle school	74 (30)
High school and higher	52 (20)
Occupation^§^	
Working on the farm	112 (45)
Housewife	40 (16)
Small scale business owner	47 (19)
Salaried	21 (9)
Others	27 (11)
Food Security Status^¶^	43 (17)
Food secure	14 (6)
Mild food insecurity	36 (14)
Moderate food insecurity	157 (63)
Severe food insecurity	
Owned a farm	32 (13)
Owned a livestock	163 (66)
Electricity in the household	235 (94)
Owned TV	139 (56)
Used wood or charcoal as a fuel	200 (80)
Mud flooring the household	148 (59)

**^†^**Time represented to and fro and queuing time at the water source per trip.

**^‡^**n = 169 participants were able to recall the amount of water available in the household at the time of interview;

^§^Other in the occupation referred to those who were not have consistency in job or were students;

^¶^It was measured using FAO- Household Food Insecurity Access Scale;

^#^SD = Standard deviation rounded to the nearest full digit;

^Δ^Percentages are rounded to the nearest full digit.

Assessment of household level food insecurity indicated that only 17% of households were food secure, while 14% and 63% reported moderate and severe levels of food insecurity, respectively (Table 3). In the case of water sources, most of the participants relied on the use of nearby streams/spring water or unimproved natural sources for household chores and/or drinking. Of the total participants, 45% reported using different sources of water for drinking and household chores. As shown in Table 3, women reported spending on average 37 ± 39 minutes per water fetching trip. At the time of the survey, about 25 ± 33 liters of water was available in participants’ homes and women reported using on average 65 ± 42 liters of water for their entire household during the preceding 24 hours.

### Item reduction analyses – WATINE-21

All statements with a > 10% non-response rate were deleted from the scale. Additionally, item difficulty indexes were computed representing the proportion of affirmative answers for each question. Acceptable values are between 30% and 80%. Item discrimination indexes were computed using the corrected point-biserial correlation coefficient of affirmative item responses to the total water insecurity score with acceptable values being scores either ≥0.86 or ≤0.14. This represented the extent to which, respondents who answered yes to a particular statement had a high overall water insecurity score. Three statements – children going late to school because of water fetching (S15), discontinued animal breeding (S18), or stopping cultivating home garden (S19) – had relatively high percentages of missing cases, 13.2%, 10.4%, and 17.6%, respectively. These same three statements also didn’t meet the minimum threshold of the item difficulty index. Thus, they were removed from the scale. Also, the item discrimination index score for the statement referring to whether satisfied with the water situation (S1) was below 0.14 indicating poor correlation with the total score, resulting in the removal of that item (Tables 1 and 2).

A reanalysis of the remaining 21 items revealed improved inter-item and item-total correlations and for the purposes of the study was labeled the WATINE-21. Then, as a next step, an exploratory factor analysis was performed to assess the multidimensionality of the scale using Direct Quartimin (oblique) and Varimax (orthogonal) rotations in order to explore the latent components of statements. The statements not meeting the adopted thresholds (factor loading of 0.40 and 0.50 respectively) or that cross-loaded (loaded more than the threshold on two or more factors) were considered for exclusion after each rotation. This meant S5, S6, S7, and S22 after Direct Oblimim and S4, S5, S6, S7, S14, S20, and S22 after Varimax. Based on the results of these two analyses, the statements S5, S6, S7, and S22 did not meet inclusion from both rotations and were removed from the scale. This led to the final version of multidimensional scale with 17 items, i.e. the WATINE-17 (Table 1 and 2).

### Reliability and validity testing – WATINE-17

The Cronbach’s Alpha coefficients were computed to assess internal consistency. For the HWINS-25, Cronbach’s Alpha value was 0.932, while for reduced scale of WATINE-17 it was 0.923 (Table 1). Hence, in both cases, Cronbach’s Alpha value was greater than 0.90 showing an excellent reliability of the scale.

Predictive validity of the WATINE-17 was assessed against household food in/security status and incidence of diarrhea in the past 4 weeks among 5 years old or younger children. There was a strong positive association between water and food insecurity scores (r = 0.492, p < 0.001). An additional analysis revealed a dose-response relationship between the two measures, i.e. water insecurity score was highest among severely food insecure women in comparison to moderately food insecure and food secure women [F (3, 246) = 22.469, p < 0.001] (Table 4). About half (44%) of the participants had young children living in their households; hence the sample size of assessing incidence of diarrhea was 110. Among these households, 19% of women said ‘yes’ that the youngest child living with them had diarrhea in the past 4 weeks. As indicated in Table 4, though marginally significant, the WATINE-17 score was higher among those who reported an incidence of diarrhea among the youngest children in the past four weeks (water insecurity score, diarrhea: Yes: 18.05 ± 9.24 vs. No: 13.26 ± 12.04, p = 0.091) (Table 4).

**Table 4.** Assessment of association between the household water insecurity experience scale (WATINE) and related indicators for validity testing in rural areas in West Cameroon-Central Africa (n = 250)**^†.^**

Indicators	Values	Sig
Predictive validity		
Food Insecurity score^‡^	r = 0.492	p < 0.001
**Food in/security status^§^**	**HWINS-17 score**	
Food Secure	7.3 ± 9.6	p < 0.001
Mildly food insecure	8.3 ± 8.2	
Moderately food insecure	16.2 ± 14.8	
Severely food insecure	25.9 ± 16.3	
**Incidence of diarrhea among children 5 years or younger^§¶^**		
No	13.26 ± 12.04	p = 0.091
Yes	18.05 ± 9.24	
**Concurrent validity^‡^**		
Amount of water drank in the past 24 hours	r = 0.045	p = 0.494
Number of times drank water in the past 24 hours	r = 0.174	p = 0.007
**Convergent Validity^‡^**		
Time spent to fetch water per trip^#^	r = 0.296	p < 0.001
Amount of water stored in the household^Δ^	r = −0.175	p = 0.023
Amount of water used in the household in the past 24 hours	r = −0.157	p = 0.016

^†^The participants were women who were the main person involved in managing water for the household.

^‡^Pearson correlation coefficient;

^§^One way ANOVA;

^¶^n = 110 households with an index child;

**^#^**Includes to and fro and queuing time.

^Δ^n = 169 participants were able to recall the amount of water available in the household at the time of interview.

Concurrent validity of the scale was assessed by comparing water insecurity score and the total intake of water and frequency of water intake in the past 24 hours among participants. There was no significant association between total intake of water and the WATINE-17 score. However, the WATINE-17 score was positively associated with frequency of water intake (r = 0.174, p = 0.007) (Tables 1 and 4).

Convergent validity was conducted to assess whether the scale reflected water access by the amount of time it took per trip in fetching water and the amount of water available in the household. A significant correlation was found between the water insecurity scores and the total time spent per trip (r = 0.296, p < 0.001). Additionally, there was a negative association between the amount of water stored in the household and water insecurity score (r = −0.175, p = 0.023). Similar results were found when assessing the WATINE-17 score with the amount of water used per household member in the past 24 hours (r = −0.157, p = 0.018).

## Discussion

The ground-up approach of several rounds of in-depth investigation of water access-related experiences and issues was useful in identifying scale items relevant for rural setting in Central Africa. Furthermore, the utilization of quantitative and statistical techniques was useful in developing a validated household-level water insecurity scale representing three key domains: psychosocial distress due to inconsistent or limited access to water, quantity of water, and water quality.

In examining the results of the WATINE-17 and other scales, water insecurity is shown to be associated with mental distress and anxiety among women. In a scale development study in Ethiopia, a positive association was found between water insecurity and mental distress [11]. Similarly, in a study in Lesotho, women reported high levels of distress when access to water was limited [12]. This indicates that the uncertainty aspect of water insecurity is associated with mental and psychosocial issues among women, which cannot be captured through standard indicators such as distance to water source or time it takes to fetch water. The results of this study indicate that a feeling of frustration, anxiety, including dispute with household members due to water is frequently experienced by women. In fact, this and other studies have found that women living in water insecure areas spend considerable amount of time each day walking long distances to water sources, have to carry full water containers back to their homes, and sometimes need to repeat the process several times to collect sufficient amounts of water for their households [10,15,32] In addition to the physical demands of this process, women reported putting their personal safety at risk in the process of securing water for their families [8,33–35]. Besides, in a scale development study in Ethiopia, a positive association was found between water insecurity and mental distress [11]. Hence, as expected, three items related to mental distress were retained in the final scale, involving being worried, upset, and having arguments due to water.

As was reported in most of the other water security scales, inability to carry out household activities due to a limited quantity of water is a prominent component of the WATINE-17. The scale includes several statements on the incapability to conduct daily chores due to lack of water, such as being able to clean toilets, wash dishes, and do laundry. An item enquiring about the changes in meal plan or cooking something different also was retained signifying the negative role that water insecurity can play on diet quality. For instance, in the previous study, it was found that water shortage involved diverting from complete meal of *Koki* (black eyed peas) and Couscous to eating raw or grilled items, such as roasted yams [8]. The scale also includes an item on the reduction in daily intake of water. This is critical since water is an essential nutrient and can affect health status significantly.

We found that water insecurity was associated with higher frequency of water intake. This positive correlation between water insecurity and frequency of water intake might be a coping strategy. Since, in the initial phase, women indicated that they drank water moreover to ‘wet the throat’ versus to drinking fully to save drinking water for other household members [8]. In the future, further investigation on how water insecurity affects daily intake of water using osmolarity status might be useful, along with the further refinements of methods to measure daily water intake.

The other major domain of WATINE-17 represented quality aspect of water. In the scale, recycling water for household chores was common. Respondents were able to relate with the statement regarding drinking muddy, unclear, or bad smelling water. Overlapping with the quantity domain, women also identified the issue of not being able to drink water because of its taste. In some of the other reported scales, water insecurity was associated with drinking bad quality water [6]. For instance, in the scale by Tsai et al. (2016) in rural Uganda, two items were included representing poor quality of drinking water, i.e. drinking from undesirable source of water and drinking unsafe water [15].

Similar to other studies findings [15,36], the formative phases of this research highlighted the primary role women played in fetching and managing water for their households. Hence, they were chosen as the sole respondents of the testing phase of the scale. Not surprisingly, the same methodology was used by the majority of water insecurity scale development researchers in the past [10,16,17]. This emphasizes the importance of women being the speakers regarding water access issues in their communities and therefore the need for increasing their participation in leadership positions involving water utilities, water supply, and irrigation-related decisions [8].

The WATINE-17 was found to have a high predictive validity for food insecurity. Specifically, a dose-response relationship was seen, indicating that high extents of water insecurity were associated with increased food insecurity, including hunger. These results highlight that the WATINE-17 is a robust, validated, and applicable scale for rural communities, where food availability is largely depended upon households’ ability to grow food. The results also suggest that water insecurity increases the risk for food insecurity through several mechanisms, including ability to grow food, clean food, and cook. Though marginally significant, the WATINE-17 was also associated with high rates of diarrhea among children, indicating the scale’s ability to capture one of the outcomes of food insecurity of poor food utilization. In the future, research assessing the relationship between the HWINS-17 and the recovery rate of children from diarrhea using oral rehydration therapy is also warranted, since clean and sufficient amount of water is critical for its effectiveness. Moreover, a focus on food safety could help exploring the effect of water insecurity in a context of direct exposure to contaminated water and/or unhygienic environment due to insufficient availability of water.

Similar to the household food insecurity scale development,, the water insecurity scale development is also occurring in a successive nature with investigators using previous studies to further refine their scales. The WATINE-17 is the latest scale, and the first instrument developed and tested in Central Africa, representing a common setting of rural areas with prevailing economic water scarcity. The WATINE-17 – household level experiential scale strengthens the existing UN-water indicators, such as distance to water source and amount of water use per capita, in monitoring and evaluating WASH-related programs and interventions. Moving forward, a joint meeting sponsored by health organizations, such as UN-Water, is warranted to review, plan and develop universal water insecurity scale to monitor and evaluate SDG# 6 and other related measures. The WATINE-17 adds to the current water insecurity measurement scale literature and highlights the inter-connections between water insecurity, nutrition, and health. Nevertheless, the WATINE-17 scale has certain limitations, including the inability to designate different levels of water insecurity. The study was able to demonstrate the correlation between water insecurity and other related indicators. However, further investigation on scoring and adapting of the scale to designate different levels of severity is warranted. Furthermore, the formative phase or original selection of quotes/items was done in French, which was then translated into English, potentially causing some translation or wording error. However, the experts review involved revisions by a bilingual person to ensure identical comparability between French and English statements. Finally, the HWINS-17 was developed in a rural setting representing economic scarcity of water. Hence, further validation and testing is warranted to establish its use in urban areas and in rural areas where rainfall is scanty and physical shortage of water is also an issue.

## Conclusions

An extensive investment in the formative phase and a rigorous field testing has led to the development of valid and reliable household-level water insecurity scale, the WATINE-17. Moving forward, more research into categorization of the HWINS-17 scoring scheme to highlight the severity levels of water insecurity is envisioned.

In the measurement of water insecurity at the household level, items enquiring about changes in household chores (quantity) and use of unclean water (quality) are critical. Validation results of the WATINE-17 emphasize a close connection between water insecurity and food insecurity. Hence, the assessment of water insecurity is not only relevant for SDG#6 of ensuring universal access to water for all, but it can also help plan and monitor other SDGs such as achieving food security (SDG#2) and promoting well-being (SDG#3). The water insecurity assessment studies have shown gender differences, with women more vulnerable to experiencing it than men. An in-depth investigation of intra-household differences in water insecurity experience will be useful to understand what role each household member plays and if there is a differential effect of water insecurity by gender and age groups. Overall, It is time to recognize the importance of water and the importance of integrated nutrition and water interventions that are desperately needed to address malnutrition in developing countries.

## Paper context

The WATINE-17 is the latest and the first water insecurity scale developed and tested in Central Africa, representing a common setting of rural areas with prevailing economic water scarcity. The methodological approach involving extensive formative phase is important in developing an experience-based scale. Validation results of WATINE-17 emphasize a close connection between water and food insecurity. The assessment of water insecurity is critical to ensure universal access to water and monitor other SDGs.
